# Correlation between Strawberry (*Fragaria ananassa* Duch.) Productivity and Photosynthesis-Related Parameters under Various Growth Conditions

**DOI:** 10.3389/fpls.2016.01607

**Published:** 2016-10-26

**Authors:** Hyo G. Choi, Byoung Y. Moon, Nam J. Kang

**Affiliations:** ^1^Department of Horticultural Science, Kongju National UniversityYesan, Korea; ^2^Department of Biological Sciences, Inje UniversityGimhae, Korea; ^3^Institute of Agriculture and Life Science, Gyeongsang National UniversityJinju, Korea

**Keywords:** chlorophyll fluorescence, greenhouse, photosynthesis, phytochemicals, pigment, shading, strawberry, temperature

## Abstract

In the present study, we investigated changes in chlorophyll fluorescence, photosynthetic parameters and fruit yields, as well as fruit phytochemical accumulation of strawberry (*Fragaria ananassa* Duch.) that had been cultivated in a greenhouse under different combinations of light intensity and temperature. In plants grown with low light (LL) photosystem II chlorophyll fluorescence was found to increase as compared with those grown under high light (HL). When strawberry plants were grown with temperature higher than 5°C in addition to LL, they showed decrease in non-photochemical quenching (NPQ), photochemical quenching (qP), as well as chlorophyll fluorescence decrease ratio (R_Fd_) when compared with other combinations of light and temperature. Moreover, fruit yield of strawberry was closely correlated with chlorophyll fluorescence-related parameters such as NPQ, qP, and R_Fd_, but not with the maximum efficiency of PS II (Fv/Fm). Although plant groups grown under different combinations of light and temperature showed almost comparable levels of photosynthesis rates (Pr) when irradiated with low-intensity light, they displayed clear differences when measured with higher irradiances. Plants grown under HL with temperature above 10°C showed the highest Pr, in contrast to the plants grown under LL with temperature above 5°C. When the stomatal conductance and the transpiration rate were measured, plants of each treatment showed clear differences even when analyzed with lower irradiances. We also found that fruit production during winter season was more strongly influenced by growth temperature than light intensity. We suggest that fruit productivity of strawberry is closely associated with chlorophyll fluorescence and photosynthesis-related parameters during cultivation under different regimes of temperature and light.

## Introduction

Unpredictable fluctuations of temperature and increased cloudy days during the cultivation seasons have been reported to negatively affect crop productions (Kucharik and Serbin, 2008; Liu et al., 2014). In particular, environmental factors such as light (Biswal and Biswal, 1999; Zivcak et al., 2014; Rapparini et al., 2015) and temperature (Lafta and Lorenzen, 1995; Gupta et al., 2016) have a significant impact on the growth and development of plants. Low temperature (LT) is one of the most significant abiotic stresses limiting plant photosynthesis (Ashraf and Harris, 2013) and severely retards plant growth and function (Gupta et al., 2016). Light intensity is also important for the growth, morphogenesis and other physiological responses of plants (Hussey, 1963; Keren et al., 1997; Ali et al., 2005; Schneider et al., 2006). Low light (LL), in particular lowers the rate of photosynthesis and thus limits the yield of crops (Lichtenthaler et al., 1981; Dong et al., 2014).

Chlorophyll fluorescence (ChlF), photosynthesis rate (Pr) and related parameters such as stomatal conductance (Sc) and transpiration rate (Tr) have been introduced in diagnosing the physiological responses of plants under abiotic stresses such as drought, low light, or salinity (Lee et al., 2001; Miyashita et al., 2005; Barbieri et al., 2012). Fv/Fm represents the maximum potential quantum efficiency of photosystem (PS II) and an Fv/Fm value in the range of 0.79 to 0.84 is optimal for many plant species, with lowered value indicating plant stress (Maxwell and Johnson, 2000). Additionally some studies explained indication of photosynthetic capacity using value of ChlF which can be represented by the rate of electron transport of PSII (Jamil et al., 2007; Ashraf and Harris, 2013). Moreover, the technique of chlorophyll fluorescence imaging is being widely used as an alternative method to accurately quantify tolerance and acclimation of leaves to environmental stresses (Ehlert and Hincha, 2008).

Productivity as well as accumulation of phytochemicals in the fruits of strawberry (*Fragaria ananassa* Duch.) are affected by various environmental conditions (Zheng et al., 2007; Choi et al., 2014, 2015). In particular, phenolic compounds not only have functional roles in plant metabolism and growth but are essential for the organoleptic qualities of the fruits (Tulipani et al., 2008).

While increasing numbers of Korean farmers tend to cultivate strawberry plants using the two-floor bench bed system in a greenhouse, they also try to reduce costs of warming the greenhouse in the winter by keeping the temperature as low as possible. Plants grown in the lower bed is usually shaded by plants growing in the upper bed because they intercept sunlight. For the optimum fruit production of strawberry, winter cultivation in the greenhouse is inevitable in Korean horticulture, but increase in the cloudy days together with capricious temperature changes during winter season tends to be an obstacle in the greenhouse agriculture. In order to address those problems, we aimed at investigating how the variations of temperature and light levels influence the greenhouse cultivation of strawberry during winter in terms of photosynthetic performances.

In the present study, we aimed at evaluating growth and productivity of strawberry plants in terms of chlorophyll fluorescence and related parameters of photosynthesis when they are cultivated under varying combinations of light intensity and temperature.

## Materials and methods

### Plant cultivation and materials

Two-floor bench bed systems consisting of upper and bottom beds (Figure S1) were used for cultivating strawberry plants in the two independent greenhouses. Plants in the bottom bed were found to receive only about 40% of the sunlight intensity incident on the upper bed because they were overshadowed by the upper bed. Fluctuations in photosynthetic active radiation of ambient lights on the two-floor bench bed in a greenhouse were continuously recorded with 10-min interval by using LI-190 quantum sensors (Licor, NE, USA). Quantum sensors were installed at the height of 20 cm above the surface of each bed and the measured values were stored in the CR 1000 data-logger (Campbell Sci. Inc., UT, USA).

Strawberry runners were planted in the two-floor bench beds when they are at the developmental stage of the 4th leaf and were cultivated from October 15, 2014 to March 31 of the following year. They were grown in coir medium hydroponically using a drip irrigation system by providing nutrient solutions of de Kreij et al. (1999) with EC between 1.0 and 1.2 dS·m^−1^ for each 2 min at a frequency of five times per day.

We prepared two independent greenhouses experiencing two different regimes of temperatures. The temperature in each greenhouse was not allowed to fall below 5 or 10°C all day long, respectively, because the heaters called as complex environmental regulator (Woosung Hitec Co. Ltd., Yangsan, Korea) began to operate automatically every time when the room temperature drops below 5 or 10°C in the respective greenhouse. Every when each temperature was raised above the level of 5 or 10°C, heater was controlled to turn off automatically, recording the minimum temperatures of each greenhouse. The temperature sensor was set up at the height of 2.5 m above the ground in the greenhouse. In this way, four combinations of growth conditions were established, consisting of upper bed maintaining above 10°C (UT), bottom bed above 10°C (BT), upper bed above 5°C (UF), and finally bottom bed above 5°C (BF). Although light intensity and temperature inside each greenhouse varied continuously in the course of the experimental period depending on the ambient weather conditions, four different treatments named as UT, BT, UF, and BF were assumed to represent high temperature plus high light, high temperature plus low light, low temperature plus high light, and low temperature plus low light, respectively. Each group of 100 plants was subjected to four different kinds of treatment, respectively, for the subsequent analyses. The production was recorded as fruit yield harvested from December to next March. All the experiments were carried out in the greenhouses constructed in the Protected Horticulture Research Institute of Korea (latitude 35°19′N and longitude 129°22′E).

### Analysis of chlorophyll fluorescence and photosynthetic parameters

Leaves of 18 to 20-day-old plants that had been excised at 7 a.m. of January 17 were sealed in the dark bottle and swiftly moved to the laboratory to minimize water stress. Chlorophyll fluorescence (ChlF) images were immediately taken using a imaging fluorometer (FluorCAM FC800, Photon Systems Instruments, Drasov, Czech) equipped with the software of quenching analysis design protocols. ChlF induction curves upon illumination of dark adapted leaves were recorded and from these induction curves, the maximum quantum use efficiency of PS II was calculated in terms of F_V_/F_M_. Other parameters of ChlF such as non-photochemical quenching (NPQ), photochemical quenching (qP), and variable chlorophyll fluorescence decline ratio (R_Fd_) were also measured by using the FluorCam system. From the light-induced curves of ChlF in dark-adapted leaves ChlF parameters were calculated (Figure S4). Changes in Fv/Fm ratio during daytime were measured in a greenhouse on the 18th of January after supplying light pulse of 3000 μmol·m^−2^·s^−1^ by using a portable fluorometer FluorPen FP 100 (Photon Systems Instruments, Drasov, Czech).

When parameters such as photosynthetic rates (Pr), stomatal conductances (Sc), and transpiration rates (Tr) were measured, intact leaves attached to plants were subjected to *in situ* analysis using a hand-held photosynthesis system (LI-6400, Licor, NE, USA) on the 16th of January (under increasing irradiances up to 1500 μmol m^−2^ s^−1^), and on the 8th of February (under 1000 μmol m^−2^ s^−1^), respectively.

### Measurement of chlorophylls and carotenoids

To measure the amounts of chlorophylls and carotenoids, leaves were harvested three times on the each 10th of January, February, and March on a monthly basis. After punching six leaf disks (1 g fresh weight) with a cork borer, they were macerated in 15 mL of acetone (containing 100 mg of CaCO_3_) with a homogenizer (PT-3100, Kinenatica AG, Switzerland). The homogenates were poured into a solvent-resistant microfuge tube, and after spinning for 5 min, the resulting supernatants were collected. The extracts were filtered, and the absorbance was measured at 661.6, 644.8, and 470 nm with a spectrophotometer (Evolution 300, Thermo Co., MA. USA) as described by Lichtenthaler (1987).

### Preparation of fruit extracts

Fully matured strawberry fruits were harvested on the 10th of each month from December to March and each 1 kg fruits from 30 plants were provided for the three repetitions of measuring phytochemicals, sugar content, and organic acids. After homogenizing the fruits, the extracts were centrifuged with the speed of 16,000 g for 30 min at 4°C (64R Centrifuge, Beckman Coulter Inc., CA, USA). Filtered supernatants through Whatman No. 2 filter paper were immediately frozen to be stored at −70°C as referred by Choi et al. (2015).

### Analysis of sugars and organic acids

Before measuring the soluble solid contents of fruit extracts, frozen samples were melted and filtered through 0.45 μm syringe filter. After diluting the filtrates with distilled water, they were analyzed with a HPLC system (YL9100, Younglin Co., Anyang, Korea) equipped with a Sugar-Pak (4.6 mm × 250 mm, Supelco, PA, USA) column and RI detector according to Choi et al. (2015).

Amounts of organic acids contained in the fruit extracts were analyzed with ion chromatography system (ICS 5000, Dionex, CA, USA) equipped with Ion-Pac column (9 × 250 mm ICE-AS6, Dionex, NY, USA) and a suppressor (AMMS ICE300, Dionex, NY, USA) according to Choi et al. (2015).

### Analysis of phytochemicals

Anthocyanin contents in the fruit extracts were measured and calculated with reference to pelargonidin-3-glucoside as the standard according to the modified procedure of Kim et al. (2011). Briefly, fruit extracts were pretreated with methanol and 1% hydrochloric acid and filtered solutions were subjected to spectrophotometric measurements of absorbance at 530 nm. The content of total phenolic compounds in the fruit extracts was determined according to Slinkard and Singleton (1977) with reference to gallic acid equivalents as the standard.

### Statistical analysis

The experimental data obtained from chlorophyll fluorescence measurements, photosynthesis parameters, photosynthetic pigments, sugar and organic acid, and phytochemical analysis were subjected to a randomized complete block design (RCBD) analysis of variance (ANOVA) using SAS (SAS Institute Inc., NC, USA). Values of *P* < 0.05 were regarded as significant.

## Results

### Changes in light and temperature experienced by plants during growth in different bed positions in the high-bench system

Strawberry plants were cultivated in the high-bench bed system composed of upper and bottom bed (Figure S1). We recorded changes in the level of greenhouse daylight incident to plants growing in the upper or bottom bed during the cultivation period (Figure S2). Since heat insulation films were covered around the greenhouse during winter for the purpose of preserving the inner temperature, they were cleared away every day from 8 a.m. to 5 p.m. so that sunlight could penetrate into the greenhouse. Overshadowed by the upper bed, the light intensity of bottom bed was sharply dropped after 11 a.m. The respective monthly average of ambient daylight integral given to the upper and the bottom bed was 10.45 and 4.38 mol·m^−2^·d^−1^ in December, 10.33 and 3.54 mol·m^−2^·d^−1^ in January, 12.84 and 4.82 mol·m^−2^·d^−1^ in February, and 19.54 and 7.35 mol·m^−2^·d^−1^ in March (Figure S2). In overall, the light intensity incident to the bottom bed was about only 40% of the upper bed.

We also recorded changes in the greenhouse temperature during the cultivation period (Figure S3). The minimum temperature of each greenhouse was controlled not to decline below 5 or 10°C during cold season. Air ventilation of the greenhouse was carried out by opening or closing the side window of the greenhouse when the inner temperature was over or below 25°C, respectively. As a result, the minimum temperature of each greenhouse was kept above 5 or 10°C, the upper temperatures were quite variable depending on the outside weather. It was noticeable that daytime temperature of the greenhouse increased significantly following the end of February.

### Chlorophyll fluorescence

**Figure 1:**
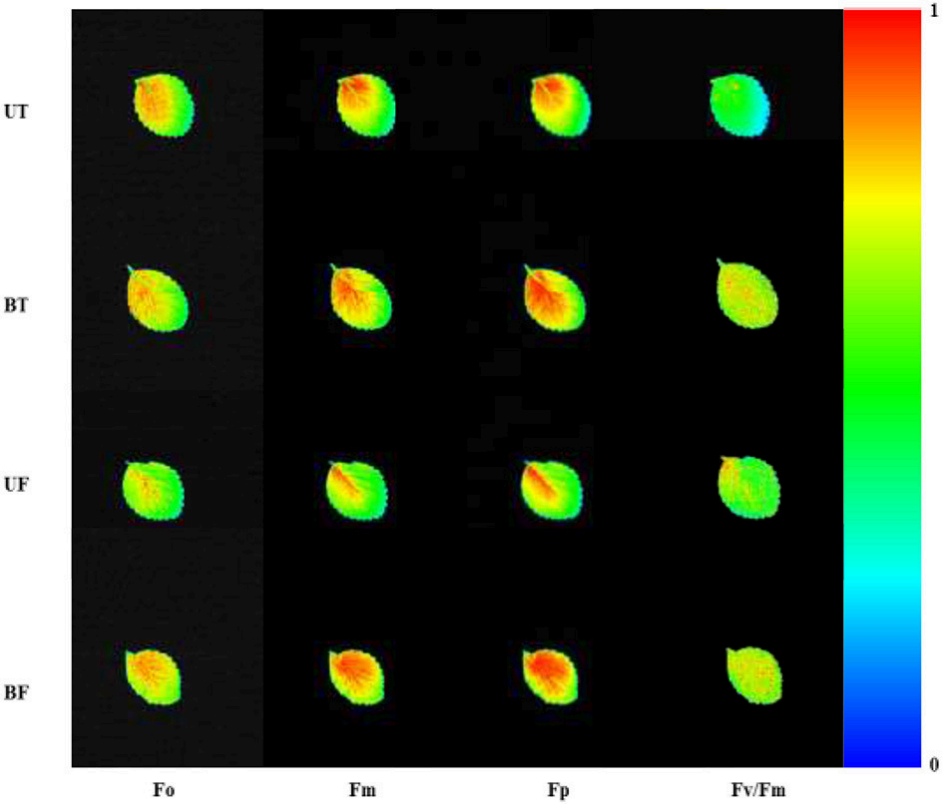
**Chlorophyll fluorescence imaging in strawberry leaves cultivated under different combinations of light and temperature regimes**. Leaves were harvested at 7:00 a.m. on January 17, 2015 and subjected to chlorophyll fluorescence induction after dark adaptation of the leaves for 20 min. BF, plants of the bottom bed under heating start temperature at 5°C; BT, plants of the bottom bed under heating start temperature at 10°C; UF, plants of the upper bed under heating start temperature at 5°C; UT, plants of the upper bed under heating start temperature at 10°C.

For plants grown under four different conditions of light and temperature consisting of UT, BT, UF, and BF, chlorophyll fluorescence image was visualized from leaves of each plant (Figure 1). BT and BF leaves of the bottom bed under low light showed higher ChlF of Fo, Fm, Fp, and Fv/Fm than UT and UF leaves of the upper bed under high light. ChlF was most strongly emitted at leaf base close to the petiole and extended to the margin of leaves. Leaves exposed to different temperatures of 5 and 10°C showed little differences in the emission of ChlF.

**Figure 2:**
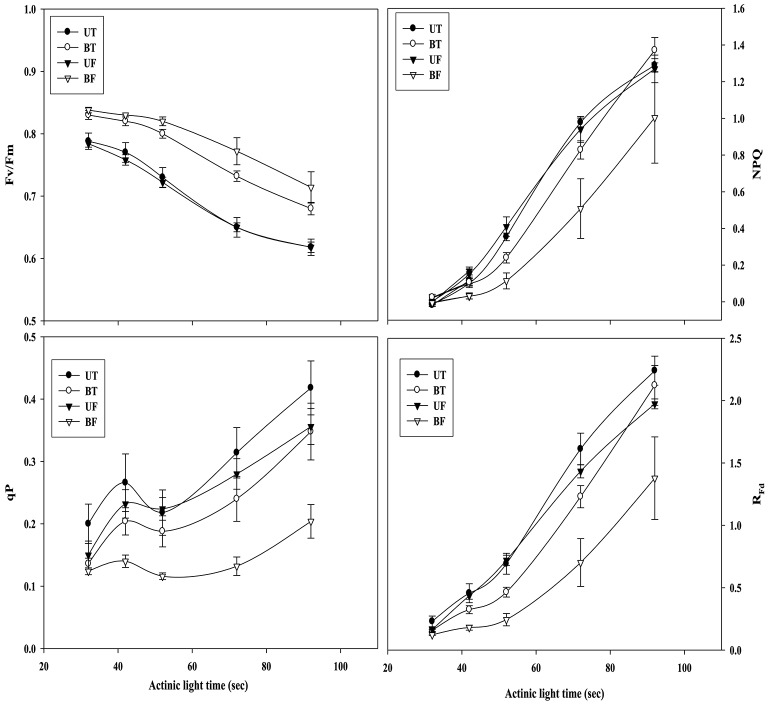
**Actinic light-dependent changes in chlorophyll fluorescence parameters of strawberry leaves during cultivation under different regimes of light and temperature**. For the induction of chlorophyll fluorescence kinetics, actinic light was provided at an intensity of 700 μmol·m^−2^·s^−1^. From each group of plants 5 sheets of leaves were harvested at 7:00 a.m. on January 17, 2015 and subjected to chlorophyll fluorescence analysis after dark adaptation for 20 min. Deviation bars indicate ± S.D. of the means. Other remarks are as shown in Figure 1.

**Figure 3:**
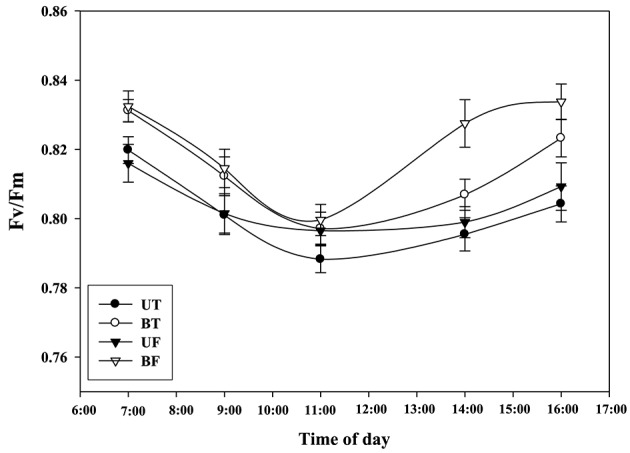
**Changes in Fv/Fm of strawberry plants over time during the day when grown under different regimes of light and temperature in a greenhouse**. For each measurement leaves from 20 individual plants were harvested on January 18, 2015 and subjected to chlorophyll fluorescence induction after dark adaptation for 20 min. Deviation bars indicate ± S.D. of the means. Other remarks are as shown in Figure 1.

Figure 2 shows time-dependent changes in several parameters of ChlF induction from leaves of strawberry when irradiated by actinic light at an intensity of 700 μmol·m^−2^·s^−1^. NPQ, qP, and R_Fd_ values obtained from BF plants were significantly lower than those values from the other plant groups except for Fv/Fm. Figure 3 shows local-time dependent changes in Fv/Fm from morning till night from leaves of plants that had been cultivated under different regimes of light and temperature. All the plants showed the highest Fv/Fm toward the sunrise, while they recorded the lowest valued around 11 a.m. when the ambient light of the greenhouse was brightest, ranging from 0.79 to 0.80 (Figure S2A; Figure 3). Generally speaking, Fv/Fm ratios measured from plants that had been cultivated under different regimes of light and temperature ranged from 0.79 to 0.84. Plants of BF showed higher Fv/Fm ratios than those of UT. When each Fv/Fm ratio was compared in terms of growth temperature, plants under low temperature (UF and BF) showed higher values between 0.81 and 0.83 than those grown under high temperature (UT and BT) with values between 0.80 and 0.82.

### Photosynthetic rate and related parameters

**Figure 4:**
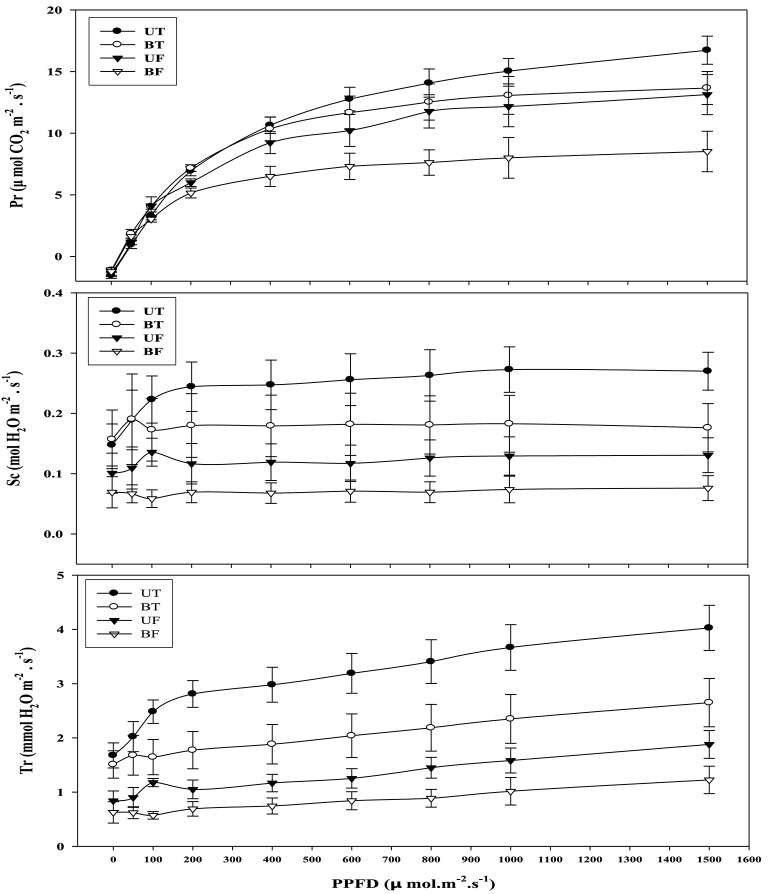
**Light-intensity dependent changes in photosynthetic rate and related parameters rates of strawberry plants during cultivation under different regimes of light and temperature in a greenhouse**. Leaves were harvested at around noon of January 16, 2015 and each supplied with increasing irradiances (PPFD) up to 1500 μmol·m^−2^·s^−1^ each for 21 min for each measurement. Each datum point represents the mean of the results obtained from each 10 individual plants. Deviation bars indicate ± S.D. of the means. Pr, photosynthetic rate; Sc, stomatal conductance; Tr, transpiration rate. Other remarks are as shown in Figure 1.

**Figure 5:**
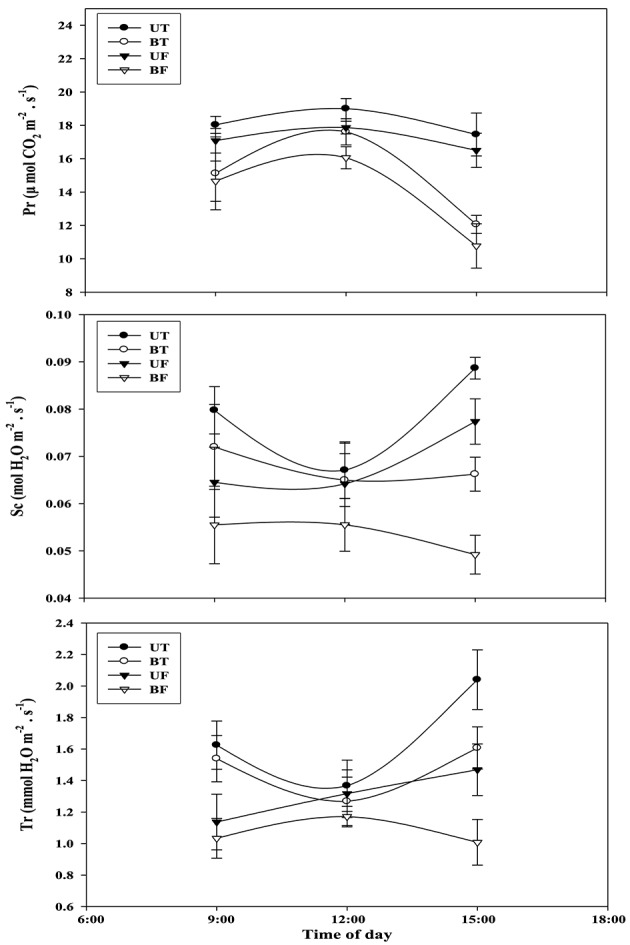
**Changes in photosynthetic rate and related parameters of strawberry plants over time during the day when plants are cultivated under different regimes of light and temperature**. For each measurement, 10 sheets of strawberry leaf were harvested on February 8, 2015 and each supplied with light at an intensity of 1000 μmol·m^−2^·s^−1^. Deviation bars indicate ± S.D. of the means. Other remarks are as shown in this figure.

Light-intensity dependent changes in photosynthetic rate (Pr) as well as related parameters such as stomatal conductance (Sc) and transpiration rate (Tr) were investigated with leaves of strawberry plants each under UT, BT, UF, and BF conditions (Figure 4). Each parameter of UT plants showed the highest level followed in turn by those of BT, UF, and BF plants. Differences in Pr between UT and other plants became clearly seen starting from the light intensity of 200 μmol·m^−2^·s^−1^ that had been irradiated for the induction of photosynthesis. When the actinic light of 800 μmol·m^−2^·s^−1^ was irradiated, UT plants showed the highest Pr value of 14.06 μmol CO_2_·m^−2^·s^−1^ as compared with 12.53 of BT, 11.77 of UF and 7.62 of BF plants, amounting to the increase of 11.2, 19.5, and 84.5%, respectively. Furthermore, BF plants recorded the lowest value of Sc and Tr, each showing 0.069 and 0.89 mmol H_2_O·m^−2^·s^−1^ as compared with other groups of plants. Changes in Pr, Sc, and Tr were investigated with the progress of local time during the daytime and the results were shown in Figure 5. At noon when the light intensity was at the peak, UT plants showed the highest value of Pr while their Sc and Tr were at the lowest level. In contrast to this, BF plants recorded the lowest levels in these parameters during the measurement, showing the least values at 3:00 pm (Figures S2, S3; Figure 5).

### Photosynthetic pigments

**Figure 6:**
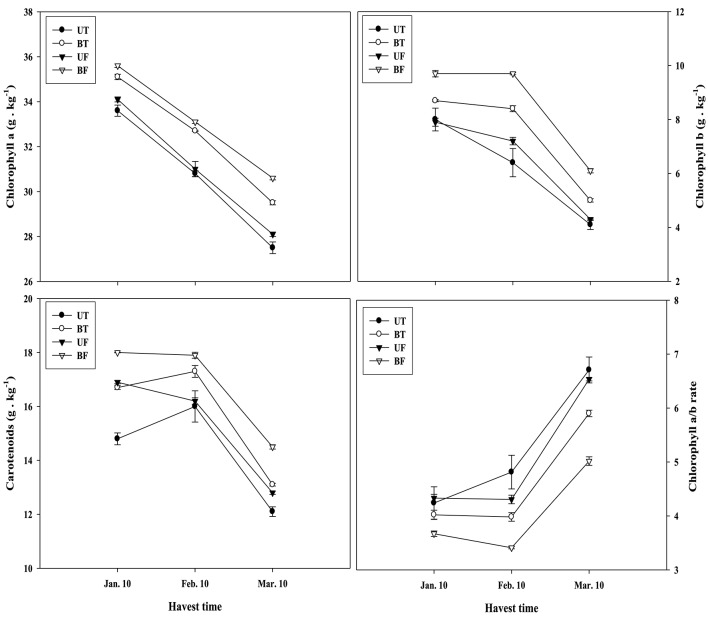
**Changes in photosynthetic pigments of strawberry plants monthly monitored during growth in a greenhouse under different regimes of light and temperature**. Measurements were done in three replicates with each replicate analyzing 100 g fresh weight of leaves collected from twenty plants. Deviation bars indicate ± S.D. of the means. Other remarks are as shown in Figure 1.

Figure 6 shows the amount of photosynthetic pigments in the leaves of UT, BT, UF, and BF plants that had been measured on a monthly basis from January to March of 2015. Amounts of chlorophyll *a, b*, and carotenoids were the highest, each showing 35.6, 9.7, and 18 g·kg^−1^, in leaves of BF plants that had been grown under lower light and temperature. While Chlorophyll *a* was shown to continuously decrease from the start of January, contents of chlorophyll *b* and carotenoids were maintained at the initial level till the start of February and thereafter began to decline. In March when the strength of sunlight enhances, it was noticed that pigments in leaves of each plant groups were markedly reduced. For example in BT plants, levels of chlorophyll a, chlorophyll b, and carotenoids decreased by 15.9, 42.6, and 21.6%, respectively. Despite such outcomes, it was evident that plant groups under higher light condition (UT and UF) accumulated less amounts of photosynthetic pigments compared with those plant groups exposed to lower light (BT and BF; Figure 6). On the contrary, the ratio of chlorophyll *a* and *b* recorded a remarkable increase from February till March, with UT plants showing the highest value of 6.7 while BF plants the lowest of 5.0 during March.

### Fruit yield

**Figure 7:**
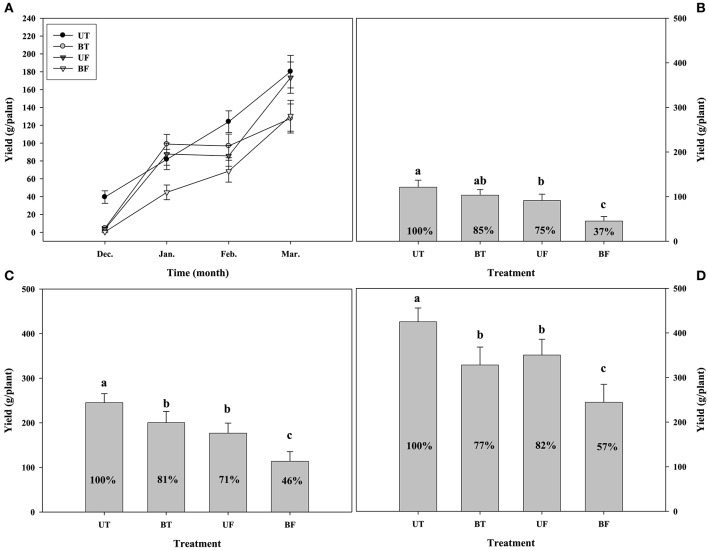
**Changes in fruit yield on a plant basis that had been monthly harvested during cultivation of strawberry plants in a greenhouse under different regimes of light and temperature**. **(A)** Monthly yield from December 2014 to March 2015; **(B)** the cumulative yield up to January 2015; **(C)** the cumulative yield up to February 2015; **(D)** the total cumulative yield up to March 2015. Each value represents the mean of 100 plants. Deviation bars indicate ± S.D. of the means. Letters above the bars indicate mean separation by Duncan's multiple range test at *p* < 0.05. Other remarks are as shown in Figure 1.

Figure 7 shows the monthly production as well as the cumulative production of strawberry fruits measured until March from December of the previous year. The respective plants of each group showed a steady increase in the fruit yield with the progress of cultivation days from December to March of the following year, with UT plants showing the highest while BF plants the lowest yield. Although January had seen a sharp increase in the fruit production of BT and UF plants compared with December, there were no differences between January and February. In contrast to this, plants of UT and BF showed almost a linear increase in the fruit production with the passage of months during the growth period. Total cumulative productions of fruits from December to March were calculated for each plant group (Figure 7D). UT plants showed the highest fruit production of 425 g per plant, followed by 350 g for UF plants, 328 g for BT plants, and 244 g for BF plants, corresponding to 82, 77, and 57% of UT plants.

### Soluble sugars and organic acids

Effects of different light and temperature on the accumulation of soluble sugars and organic acids in the strawberry fruits are shown in Tables 1, 2. Total sugars of the fruits harvested in March declined by 29, 51, 47, and 64% lower than those in January for the plants of UT, BT, UF, and BF, respectively. On the contrary, organic acids of fruits harvested in March were elevated by 36, 36, 16, and 23% higher than those in January for the plants of UT, BT, UF, and BF, respectively. The decreased extent of fruit sugars was found to be much larger than the increased extent of organic acids in the fruits as the cultivation continues. In particular, fruits of UT and UF had higher capacity to build total soluble sugar and organic acids than those of BT and BF.

**Table 1:** **Contents of soluble sugars in the fruits of strawberry that had been cultivated under different environmental conditions**.

**Treatment**	**Fructose (g·100 g^−1^, FW)**			**Glucose (g 100 g^−1^, FW)**			**Sucrose (g 100 g^−1^, FW)**			**Total sugars (g 100 g^−1^, FW)**		
	**Jan. 10**	**Feb. 10**	**Mar. 10**	**Jan. 10**	**Feb. 10**	**Mar. 10**	**Jan. 10**	**Feb. 10**	**Mar. 10**	**Jan. 10**	**Feb. 10**	**Mar. 10**
UT	1.99 ± 0.12a^z^	1.77 ± 0.16a	1.42 ± 0.28a	1.84 ± 0.18a	1.54 ± 0.08a	1.29 ± 0.24a	5.48 ± 0.35a	3.84 ± 0.18a	3.88 ± 0.92a	9.31 ± 0.58a	7.15 ± 0.18a	6.61 ± 1.42a
BT	2.03 ± 0.11a	1.77 ± 0.09a	0.79 ± 0.06b	1.91 ± 0.12a	1.62 ± 0.25a	0.64 ± 0.06b	4.14 ± 0.30b	3.75 ± 0.05a	2.53 ± 0.49b	8.08 ± 0.53a	7.14 ± 0.29a	3.96 ± 0.50b
UF	2.08 ± 0.17a	1.68 ± 0.04a	0.78 ± 0.12b	2.03 ± 0.19a	1.58 ± 0.16a	0.68 ± 0.10b	5.18 ± 0.50ab	3.55 ± 0.20a	2.98 ± 0.46b	9.31 ± 0.86a	6.87 ± 0.28a	5.02 ± 0.42b
BF	2.13 ± 0.10a	1.69 ± 0.06a	0.71 ± 0.05b	2.03 ± 0.13a	1.55 ± 0.20a	0.58 ± 0.03b	4.73 ± 1.0ab	3.63 ± 0.20a	2.56 ± 0.45b	8.91 ± 0.83a	6.82 ± 0.26a	3.28 ± 0.44b

z *Average values and standard deviation of 3 replicates were presented, each repeat being tested with 1 kg of fruits collected from 30 individual plants. Different letters indicate statistical differences within columns by Duncan's multiple range test at p < 0.05*.

**Table 2:** **Contents of organic acids in the fruits of strawberry that had been cultivated under different environmental conditions**.

**Treatment**	**Citric acid (mg 100 g^−1^, FW)**			**Malic acid (mg 100 g^−1^, FW)**			**Acetic acid (mg ·100 g^−1^, FW)**			**Total acids (mg 100 g^−1^, FW)**		
	**Jan. 10**	**Feb. 10**	**Mar. 10**	**Jan. 10**	**Feb. 10**	**Mar. 10**	**Jan. 10**	**Feb. 10**	**Mar. 10**	**Jan. 10**	**Feb. 10**	**Mar. 10**
UT	426 ± 16a^z^	529 ± 13a	584 ± 2a3	207 ± 12a	191 ± 25ab	263 ± 30a	10.2 ± 1.4b	16.9 ± 5.6b	16.0 ± 2.3a	643 ± 24ab	737 ± 29a	863 ± 29a
BT	369 ± 22b	461 ± 53ab	487 ± 11b	205 ± 12a	214 ± 10a	291 ± 10a	7.5 ± 0.7b	48.2 ± 1.3a	15.0 ± 3.6a	581 ± 36b	723 ± 48a	792 ± 16b
UF	451 ± 15a	483 ± 64ab	493 ± 21b	239 ± 36a	196 ± 27ab	287 ± 22a	15.3 ± 0.4a	11.3 ± 4.9b	10.8 ± 0.8a	679 ± 29a	690 ± 21ab	791 ± 41b
BF	390 ± 45ab	422 ± 18b	468 ± 26b	218 ± 28a	174 ± 10b	281 ± 10a	10.3 ± 4.7a	40.3 ± 2.6ab	13.7 ± 4.9a	618 ± 78ab	636 ± 28b	763 ± 36b

z *Average values and standard deviation of 3 replicates were presented, each repeat being tested with 1 kg of fruits collected from 30 individual plants. Different letters indicate statistical differences within columns by Duncan's multiple range test at p < 0.05*.

### Phenolic compounds and anthocyanins

**Figure 8:**
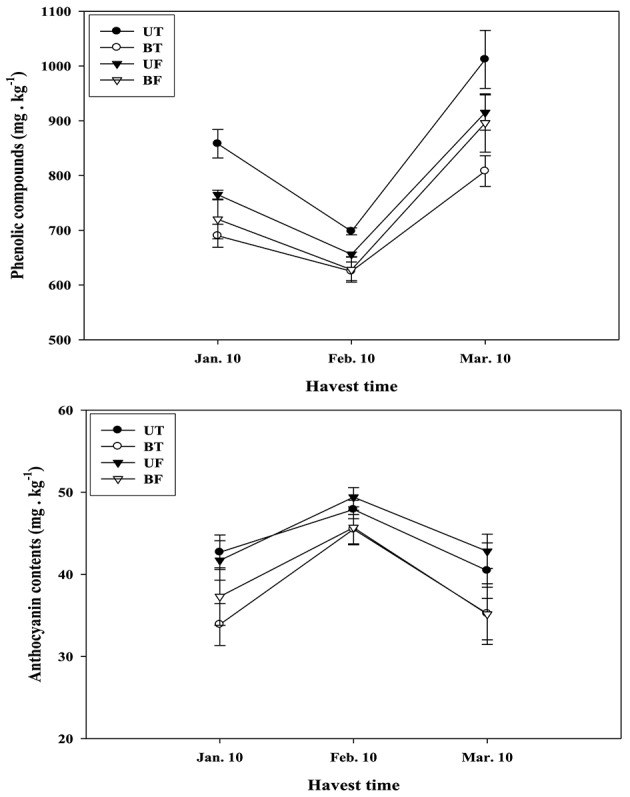
**Changes in the amounts of phytochemicals contained in the strawberry fruits that had been harvested on a monthly basis during cultivation in a greenhouse under different regimes of light and temperature**. Each value represents the mean of three replicates, with each replicate analyzing 1 kg fresh weight of fruits collected from thirty plants. Deviation bars indicate ± S.D. of the means. Other remarks are as shown in Figure 1.

Effects of different regimes of light and temperature on the formation of phenolic compounds and anthocyanins in the strawberry fruits are shown in Figure 8. Fruits of UT and UF were found to accumulate much higher amounts of phenolic compounds and anthocyanins than those of BT and BF. When changes in the amounts of the compounds were investigated on a monthly basis from January to March, it was noted that phenolic compounds in the fruits decreased from January to February followed by a sharp increase from February to March. On the contrary, anthocyanin contents were found to be significantly elevated from January to February, followed by an equivalent decrease from February to March.

### The correlation between fruit yield and photosynthesis-related parameters

The correlation analysis was carried out between fruit production and a variety of photosynthesis-related parameters, by calculating Pearson Product Moment Correlation (Pearson, 1895). As shown in Table 3, we found that fruit productions were closely correlated with Pr (*r* = 0.962, *p* < 0.01) and R_fd_ (*r* = 0.952, *p* < 0.01), showing a little less correlation with Sc (*r* = 0.874, *p* < 0.01), Tr (*r* = 0.837, *p* < 0.001) and NPQ (*r* = 0.700, *p* < 0.001). In contrast, they were negatively correlated with Fv/Fm (*r* = −0.735, *p* < 0.01).

**Table 3:** **Combined correlation coefficients between fruit yield and chlorophyll fluorescence parameters as well as photosynthetic parameters of strawberry plants**.

**Index**	**Fruit yield**	**Rfd**	**qP**	**NPQ**	**Fv/Fm**	**Pr**	**Sc**	**Tr**
Fruit yield	1.000							
Rfd	0.952^**^	1.000						
qP	0.605^*^	0.531^*^	1.000					
NPQ	0.700^**^	0.830^**^	0.053	1.000				
Fv/Fm	−0.735^**^	−0.673^**^	−0.412	−0.417	1.000			
Pr	0.962^**^	0.902^**^	0.599^*^	0.644^**^	−0.650^**^	1.000		
Sc	0.874^**^	0.794^**^	0.736^**^	0.451	−0.536^*^	0.929^**^	1.000	
Tr	0.837^**^	0.779^**^	0.740^**^	0.423	−0.491	0.868^**^	0.962^**^	1.000

*,** *Significant correlations at 5% and at 1% level (n = 16), respectively, by using Pearson correlation coefficients*.

When correlation coefficients were calculated between photosynthesis rate (Pr) and related parameters, Pr showed a significant positive correlation with Sc (*r* = 0.929, *p* < 0.01) and R_fd_ (*r* = 0.902, *p* < 0.01), with little lower correlation with Tr (*r* = 0.869, *p* < 0.01) and NPQ (*r* = 0.644, *p* < 0.01). In contrast, Pr was negatively correlated with Fv/Fm (*r* = −0.650, *p* < 0.01). R_fd_ was also positively correlated with NPQ, Sc, and Tr showing the respective correlation coefficient of 0.830 (*p* < 0.01), 0.794 (*p* < 0.01), and 0.779 (*p* < 0.01). Correlation coefficient between Sc and Tr was 0.962(*p* < 0.01) and those between qP and Sc as well as Tr were 0.736 (*p* < 0.01) and 0.740 (*p* < 0.01), respectively. The results indicated that fruit yield of strawberry was strongly dependent on photosynthesis and other related parameters.

## Discussion

**Figure 9:**
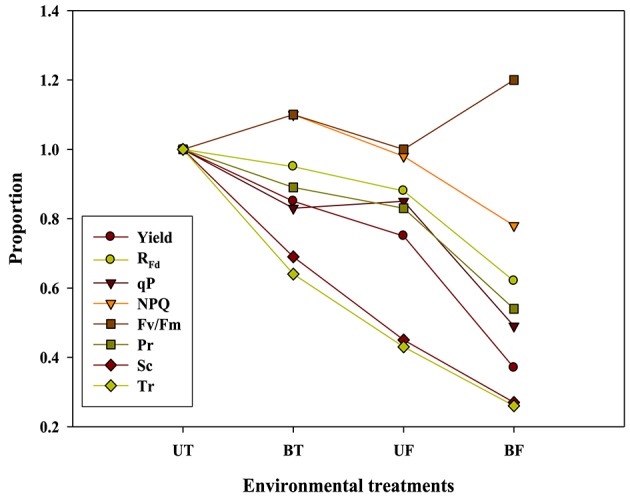
**The relationship of the graph pattern between strawberry yield and each parameter**. When UT value was represented as 1 in various parameters, BT, UF, and BF were indicated by proportion in comparison with UT. Yield value was calculated from Figure 7B; R_Fd_, qP, and NPQ values were calculated from the value measured 92 s actinic light time of Figure 2; Pr, Sc, and Tr values were calculated from the 800 μmol·m^−2^·s^−1^ of Figure 4.

Plants under stressful conditions can be diagnosed by analyzing chlorophyll fluorescence imaging (Lichtenthaler and Miehé, 1997). ChlF imaging was successfully utilized to determine freezing tolerance of Arabidopsis leaves in which freezing-induced inactivation of photosynthesis were closely monitored (Ehlert and Hincha, 2008). In the present study, when ChlF emission images from plants of UT, BT, UF, and BF were compared, it was noted that leaves of BT and BF plants showed higher PS II fluorescence than those of UT and UF plants. The results indicated that plants grown in the bottom beds were less able to carry out photosynthesis probably because they received less light by overshadowing of the upper bed. Moreover, plants of UF showed a slightly higher ChlF than those of UT (Figure 5), suggesting that lower temperature had depressed photosynthesis slightly. Fv/Fm is a normalized ratio created by dividing variable ChlF by maximum ChlF and represents the maximal efficiency of PSII to photochemistry (Baker, 2008). While Fv/Fm values in the range of 0.79–0.84 are regarded optimal for many plant species, decreased values are frequently observed when plants are exposed to various stress conditions such as high light, chilling, and salinity (Jamil et al., 2007; Na et al., 2014; Rapparini et al., 2015). In this study, it was noted that strawberry plants grown under BF condition of low light and low temperature showed higher Fv/Fm ratios than those plants grown under UT, UF, and BT conditions (Figures 2, 3). The results suggested that BF plants were in a less photoinhibited state even under low temperature probably because they were subjected to low light intensity. On the other hand, UT plants recorded higher values of NPQ, qP, and R_Fd_ than UF, BT, and BF plants (Figure 2), indicating that they had higher potential of performing photosynthesis. ChlF kinetic parameters of NPQ, qP, and R_Fd_ are also useful in measuring plant stresses under adverse environmental conditions (Lichtenthaler and Miehé, 1997; Lichtenthaler and Burkart, 1999; Zivcak et al., 2014). In consistent with the observation that R_Fd_ is an indicator correlating with the photosynthetic activity of whole leaves (Lichtenthaler et al., 2005). When the obtained data values of chlorophyll fluorescence and photosynthesis as well as of related parameters were plotted on the basis of four different growth conditions (UT, BT, UF, and BF), trends of change in Pr and cumulative fruit yield showed close association qP and R_Fd_ (Figure 9). We also calculated Pearson correlation coefficients between those parameters and clarified that there were significant positive correlation between fruit yield and parameters of chlorophyll fluorescence as well as of related parameters (Table 3). These results suggested that those parameters could be applied to predict strawberry productivity under a variety of environmental conditions. It was noted that parameters of Pr and R_Fd_, in particular, showed the highest correlation with fruit production, emerging themselves as promising indicators of plant productivity.

It was previously demonstrated that the increase of growth temperature from 5 to 10°C resulted in the promotion of rubisco activity and CO_2_ assimilation of several plant species (Sage and Sharkey, 1987). The present study showed that strawberry leaves grown under BF or UF condition had lower CO_2_ fixation than those grown under BT or UT conditions. In January when the intensity of ambient light becomes weaker, a rise in the growth temperature rather than light played a pivotal role in the promotion of photosynthetic rate (Figure 4). When CO_2_ assimilation of whole leaves was examined, plants of BT and BF showed a rapid decline around 3:00 p.m. probably due to shortage of light rather than changes in stomatal conductance or transpiration rate (Figure S2; Figure 5). In January or February when the ambient light gets weaker, stomatal conductance and transpiration rate were found to be significantly dependent on the changes in the growth temperature.

Amounts of photosynthetic pigments markedly declined during the course of cultivation (Figure 6). Chlorophyll *a* content showed almost a linear decline from January to March, while chlorophyll *b* and carotenoid content began to decrease from February. When compared between plants grown under different conditions, plants of BT and BF grown under lower light showed a higher content of these pigments relatively to those of UT and UF grown under higher light (Figure S2; Figure 6), indicating that light intensity is more important than the growth temperature in the production of photosynthetic pigments. An earlier work done by Lichtenthaler and Burkart (1999) showed that plants grown under high light had lower amount of chlorophylls per chloroplast, high chlorophyll a/b ratio, and high CO_2_ assimilation rate. They also reported that the production of chloroplast starch was greatly increased in the plants grown under sunlight as compared with those grown under shading.

When the fruit production of strawberry was compared between plants grown under different environmental conditions, the highest yield at the end of cultivation was observed in the plants of UT, sequentially followed by those of UF, BT, and BF (Figure 7). In other words, plants grown under higher light conditions showed greater fruit production than those grown under lowered light. The results indicate that the light intensity serves a crucial role in the production of fruits, probably resulting from the higher photosynthetic performances displayed by plants grown under higher light.

During January and February when ambient light intensity is lowered, there was a temporary elevation of fruit production in plants of BT under lower light as compared with those of UF under higher light (Figures 7B,C). In those periods when light intensity remains weaker and the average of ambient daylight integral was less than 13 mol·m^−2^·d^−1^ up to February, the growth temperature was found to have exerted a small but clear influence on the fruit production. Entering into March when the daylight integral was rapidly increased over 19 mol·m^−2^·d^−1^, light intensity began to exercise a stronger influence on the fruit production (Figure S2; Figure 7). When fruit production was compared between plants of BT and BF, both grown under lower light, BT plants showed a much more production than BF plants. The results suggested that in the presence of the same light intensity a rise in the growth temperature could promote the fruit production.

When the contents of soluble sugars as well as organic acids were examined in the fruits harvested at the end of the cultivation, UT and UF plants showed a higher amount than BT and BF plants. Here again, light intensity was more important than the growth temperature in the accumulation of these metabolites (Tables 1, 2). Contents of phytochemicals in the fruits were also significantly dependent on the changes in the ambient light intensity of the greenhouse.

When the photosynthetic rates and related parameters measured from strawberry plants were plotted against different combinations of light and temperature during cultivation, it became evident that fruit productivity was correlated with qP, R_Fd_, NPQ, Pr, Sc, and Tr. On the other hand, Fv/Fm ratio was found to have negative correlation with fruit yield (Table 3; Figure 9). Moreover, we came to realize that even during winter season when the ambient light grows weaker and the average daylight integral falls below 13 mol·m^−2^·d^−1^, increasing the growth temperature resulted in the promotion of photosynthetic performances followed by further improvement of fruit production. Those findings are expected to possibly give a new insight regarding the operation of strawberry industry during winter season.

## Author contributions

HC planned the study, conducted the experiment, analyzed the data, and wrote the first draft of the manuscript. BM revised the analysis of the data and edited the manuscript. NK involved within statistics section and modified the manuscript.

### Conflict of interest statement

The authors declare that the research was conducted in the absence of any commercial or financial relationships that could be construed as a potential conflict of interest.

## Abbreviations

BF: bottom bed of the greenhouse kept at temperatures not lower than 5°C
BT: bottom bed of the greenhouse kept at temperatures not lower than 10°C
ChlF: chlorophyll fluorescence
dS m^−1^: deci Siemens per meter
EC: electrical conductivity
Fv/Fm: ratio of variable fluorescence to maximal fluorescence indicating maximum quantum yield of photosystem II photochemistry in the dark-adapted state
HL: high light
HT: high temperature
LL: low light
LT: low temperature
Pr: photosynthetic rate
PS II: photosystem II
R_Fd_: variable chlorophyll fluorescence decrease ratio
Sc: stomatal conductance
Tr: transpiration rate
UF: upper bed of the greenhouse kept at temperatures not lower than 5°C
UT: upper bed of the greenhouse kept at temperatures not lower than 10°C.

## References

[B1] Ali M. B. Hahn E. J. Paek K. Y. (2005). Effects of light intensities on antioxidant enzymes and malondialdehyde content during short-term acclimatization on micropropagated *Phalaenopsis* plantlet. Environ. Exper. Bot. 54, 109–120. 10.1016/j.envexpbot.2004.06.005

[B2] Ashraf M. Harris P. J. C. (2013). Photosynthesis under stressful environments: an overview. Photosynthetica 51, 163–190. 10.1007/s11099-013-0021-6

[B3] Baker N. R. (2008). Chlorophyll fluorescence: a probe of photosynthesis *in vivo*. Annu. Rev. Plant Biol. 59, 89–113. 10.1146/annurev.arplant.59.032607.092759 18444897

[B4] Barbieri G. Vallone S. Orsini F. Paradiso R. De Pascale S. Zakharov F. N.. (2012). Stomatal density and metabolic determinants mediate salt stress adaptation and water use efficiency in basil (*Ocimum basilicum* L.). J. Plant Physiol. 169, 1737–1746. 10.1016/j.jplph.2012.07.001 22840325

[B5] Biswal B. Biswal U. C. (1999). Photosynthesis under stress: stress signals and adaptive response of chloroplasts, in Handbook of Plant and Crop Stress, ed Pessarakli M. (New York, NY: Marcel Dekker, Inc.), 315–336.

[B6] Choi H. G. Moon B. Y. Kang N. J. (2015). Effects of LED light on the production of strawberry during cultivation in a plastic greenhouse and in a growth chamber. Sci. Hort. 189, 22–31. 10.1016/j.scienta.2015.03.022

[B7] Choi H. G. Moon B. Y. Kang N. J. Kwon J. K. Bekhzod K. Park K. S. (2014). Yield loss and quality degradation of strawberry fruits cultivated under the deficient insolation conditions by shading. Hort. Environ. Biotechnol. 55, 263–270. 10.1007/s13580-014-0039-0

[B8] de Kreij C. Voogt W. Baas R. (1999). Nutrient Solution and Water Quality for Soilless Cultures. Brochure 196. Research Station for Floriculture and glasshouse vegetables (PBG) Naaldwijk.

[B9] Dong C. Fu Y. Liu G. Liu H. (2014). Low light intensity effects on the growth, photosynthetic characteristics, antioxidant capacity, yield and quality of wheat (*Triticum aestivum* L.) at different growth stages in BLSS. Adv. Space Res. 53, 1557–1566. 10.1016/j.asr.2014.02.004

[B10] Ehlert B. Hincha D. K. (2008). Chlorophyll fluorescence imaging accurately quantifies freezing damage and cold acclimation responses in Arabidopsis leaves. Plant Methods 4:12. 10.1186/1746-4811-4-12 18505561 PMC2430023

[B11] Gupta S. M. Agarwal A. Dev B. Kumar K. Prakash O. Arya M. C. (2016). Assessment of photosynthetic potential of indoor plants under cold stress. Photosynthetica 54, 138–142. 10.1007/s11099-015-0173-7

[B12] Hussey G. (1963). Growth and development in the young tomato: I. The effect of temperature and light intensity on growth of the shoot apex and leaf primordia. *J. Exp*. Bot. 14, 316–325.

[B13] Jamil M. Rehman S. Lee K. J. Kim J. M. Kim H. Rha E. S. (2007). Salinity reduced growth PS2 photochemistry and chlorophyll content in radish. Sci. Agr. 64, 111–118. 10.1590/S0103-90162007000200002

[B14] Keren N. Berg A. van Kan P. J. M. Levanon H. Ohad I. (1997). Mechanism of photosystem II photoinactivation and DI protein degradation at low light: the role of back electron flow. Proc. Natl. Acad. Sci. U.S.A. 94, 1579–1584. 11038602 10.1073/pnas.94.4.1579 PMC19834

[B15] Kim S. K. Bae R. N. Chun C. H. (2011). Changes in bioactive compounds contents of ‘Maehyang’ and ‘Seolhyang’ strawberry fruits by UV light illumination. Kor. J. Hortic. Sci. Technol. 29, 172–180. Available online at: http://www.dbpia.co.kr/Article/NODE01649835

[B16] Kucharik C. J. Serbin S. P. (2008). Impacts of recent climate change on Wisconsin corn and soybean yield trends. Environ. Res. Lett. 3:034003 10.1088/1748-9326/3/3/034003

[B17] Lafta A. M. Lorenzen J. H. (1995). Effect of high temperature on plant growth and carbohydrate metabolism in potato. Plant Physiol. 109, 637–643. 12228617 10.1104/pp.109.2.637 PMC157630

[B18] Lee H. Y. Hong Y. N. Chow W. S. (2001). Photoinactivation of photosystem II complexes and photoprotection by non-functional neighbours in *Capsicum annuum* L. leaves. Planta 212, 332–342. 10.1007/s004250000398 11289597

[B19] Lichtenthaler H. K. (1987). Chlorophylls and carotenoids: pigments of photosynthetic biomembranes. Methods Enzymol. 148, 351–382. 10.1016/0076-6879(87)48036-1

[B20] Lichtenthaler H. K. Burkart S. (1999). Photosynthesis and high light stress. Bulg. J. Plant Physiol. 25, 3–16. 10.5539/jas.v7n6p69

[B21] Lichtenthaler H. K. Buschmann C. Döll M. Fietz H. J. Bach T. Kozel U.. (1981). Photosynthetic activity, chloroplast ultrastructure, and leaf characteristics of high-light and low-light plants and of sun and shade leaves. Photosynth. Res. 2, 115–141. 10.1007/BF00028752 24470202

[B22] Lichtenthaler H. K. Buschmann C. Knapp M. (2005). How to correctly determine the different chlorophyll fluorescence parameters and the chlorophyll fluorescence decrease ratio R_Fd_ of leaves with the PAM fluorometer. Photosynthetica 43, 379–393. 10.1007/s11099-005-0062-6

[B23] Lichtenthaler H. K. Miehé J. A. (1997). Fluorescence imaging as a diagnostic tool for plant stress. Trends Plant Sci. 2, 316–320. 10.1016/S1360-1385(97)89954-2

[B24] Liu Q. H. Wu X. Chen B. C. Ma J. Q. Gao J. (2014). Effects of low light on agronomic and physiological characteristics of rice including grain yield and quality. Rice Sci. 21, 243–251. 10.1016/S1672-6308(13)60192-4

[B25] Maxwell K. Johnson G. N. (2000). Chlorophyll fluorescence-a practical guide. J. Exp. Bot. 51, 659–668. 10.1093/jxb/51.345.659 10938857

[B26] Miyashita K. Tanakamaru S. Maitani T. Kimura K. (2005). Recovery responses of photosynthesis, transpiration, and stomatal conductance in kidney bean following drought stress. Environ. Exp. Bot. 53, 205–214. 10.1016/j.envexpbot.2004.03.015

[B27] Na Y. W. Jeong H. J. Lee S. Y. Choi H. G. Kim S. H. Rho I. R. (2014). Chlorophyll fluorescence as a diagnostic tool for abiotic stress tolerance in wild and cultivated strawberry species. Hort. Environ. Biotechnol. 55, 280–286. 10.1007/s13580-014-0006-9

[B28] Pearson K. (1895). Notes on regression and inheritance in the case of two parents. Proc. R. Soc. Lond. 58, 240–242.

[B29] Rapparini F. Neri L. Mihailova G. Petkova S. Georgieva K. (2015). Growth irradiance affects the photoprotective mechanisms of the resurrection angiosperm *Haberlea rhodopensis* Friv. in response to desiccation and rehydration at morphological, physiological and biochemical levels. Environ. Exp. Bot. 113, 67–79. 10.1016/j.envexpbot.2015.01.007

[B30] Sage R. F. Sharkey T. D. (1987). The effect of temperature on the occurrence of O_2_ and CO_2_ insensitive photosynthesis in field grown plants. Plant Physiol. 84, 658–664. 16665498 10.1104/pp.84.3.658 PMC1056646

[B31] Schneider S. Ziegler C. Melzer A. (2006). Growth towards light as an adaptation to high light conditions in *Chara* branches. New Phytol. 172, 83–91. 10.1111/j.1469-8137.2006.01812.x 16945091

[B32] Slinkard K. Singleton V. L. (1977). Total phenol analysis; automation and comparison with manual methods. *Am. J. Enol*. Viticult. 28, 49–55.

[B33] Tulipani S. Mezzetti B. Capocasa F. Bompadre S. Beekwilder J. Ric-devos C. H.. (2008). Antioxidants, phenolic compounds, and nutritional quality of different strawberry genotypes. J. Agric. Food Chem. 56, 696–704. 10.1021/jf0719959 18211027

[B34] Zheng Y. Wang S. Y. Wang C. Y. Zheng W. (2007). Changes in strawberry phenolics, anthocyanins, and antioxidant capacity in response to high oxygen treatments. LWT Food Sci. Technol. 40, 49–57. 10.1016/j.lwt.2005.08.013

[B35] Zivcak M. Brestic M. Kalaji H. M. Govindjee (2014). Photosynthetic responses of sun- and shade-grown barley leaves to high light: is the lower PSII connectivity in shade leaves associated with protection against excess of light. Photosynth. Res. 11, 339–354. 10.1007/s11120-014-9969-8 PMC3923118 24445618

