# Supplementary material for: Correlation between Strawberry (Fragaria ananassa Duch.) Productivity and Photosynthesis-Related Parameters under Various Growth Conditions
Source: Front Plant Sci. 2016 Oct 26;7:1607. doi: 10.3389/fpls.2016.01607 (PMC5080357; doi:10.3389/fpls.2016.01607)
Supplement: Supplementary file 1 [file DataSheet1.docx]

**SUPPLEMENTARY FIGUREs**

**
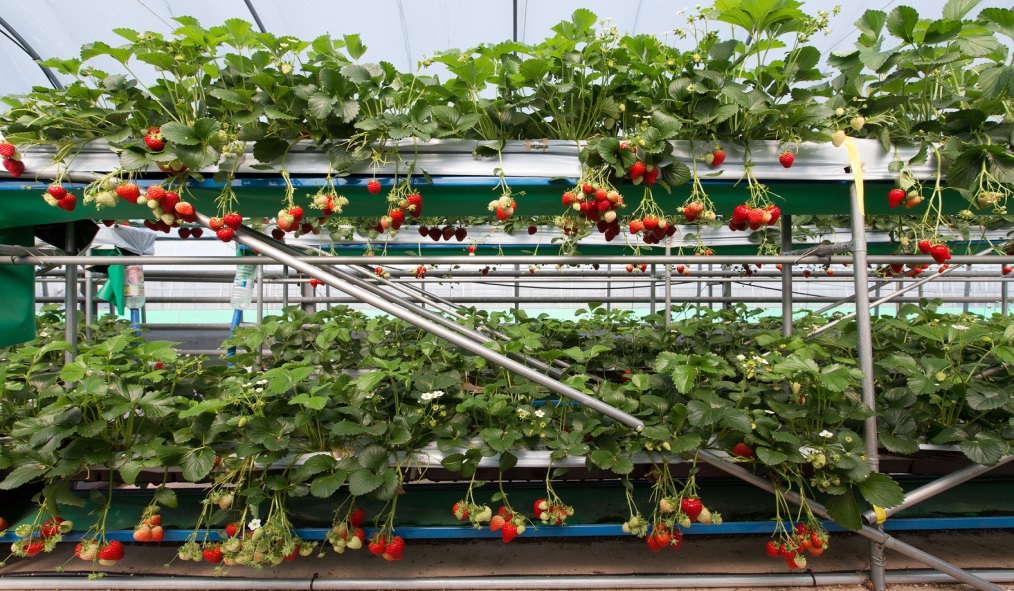
**

**SUPPLEMENTARY FIGURE S1.** Strawberry plants under cultivation on the two-floor bench bed system. Plants of bottom beds were shaded by structures of upper beds and received about 60% weaker light as compared with the plants of upper beds.

**SUPPLEMENTARY FIGURE S2.** Changes in the light intensity of the greenhouse with progression of cultivation days. A: Changes in the light intensity monitored on a sunny day of January 10, 2015; B: Changes in the light intensity monitored on a cloudy day of January 14, 2015; C: Changes in the ambient daylight integral during the month of December 2014; D: Changes in the ambient daylight integral during the month of January 2015; E: Changes in the ambient daylight integral during the month of February 2015; F: Changes in the ambient daylight integral during the month of March 2015.

**SUPPLEMENTARY FIGURE S3.** Monitoring of temperature changes in the greenhouse during the cultivation of strawberry plants. A: Time-dependent temperature variation in the greenhouse during the clear day of January 10; B: Time-dependent temperature variation in the greenhouse during the cloudy day of January 14; C: Daily changes in the greenhouse temperature during December, 1014; D: Daily changes in the greenhouse temperature during January, 1015; E: Daily changes in the greenhouse temperature during February, 1015; F: Daily changes in the greenhouse temperature during March, 1015; ● represents the change in the minimum temperatures in the greenhouse when heating was started below 5◦C; ○ represents the change in the minimum temperatures when heating was started below 10◦C; ▼ represents the change in the maximum temperatures when heating was started below 5◦C; ▽ represents the change in the maximum temperatures when heating was started below 10◦C.

**
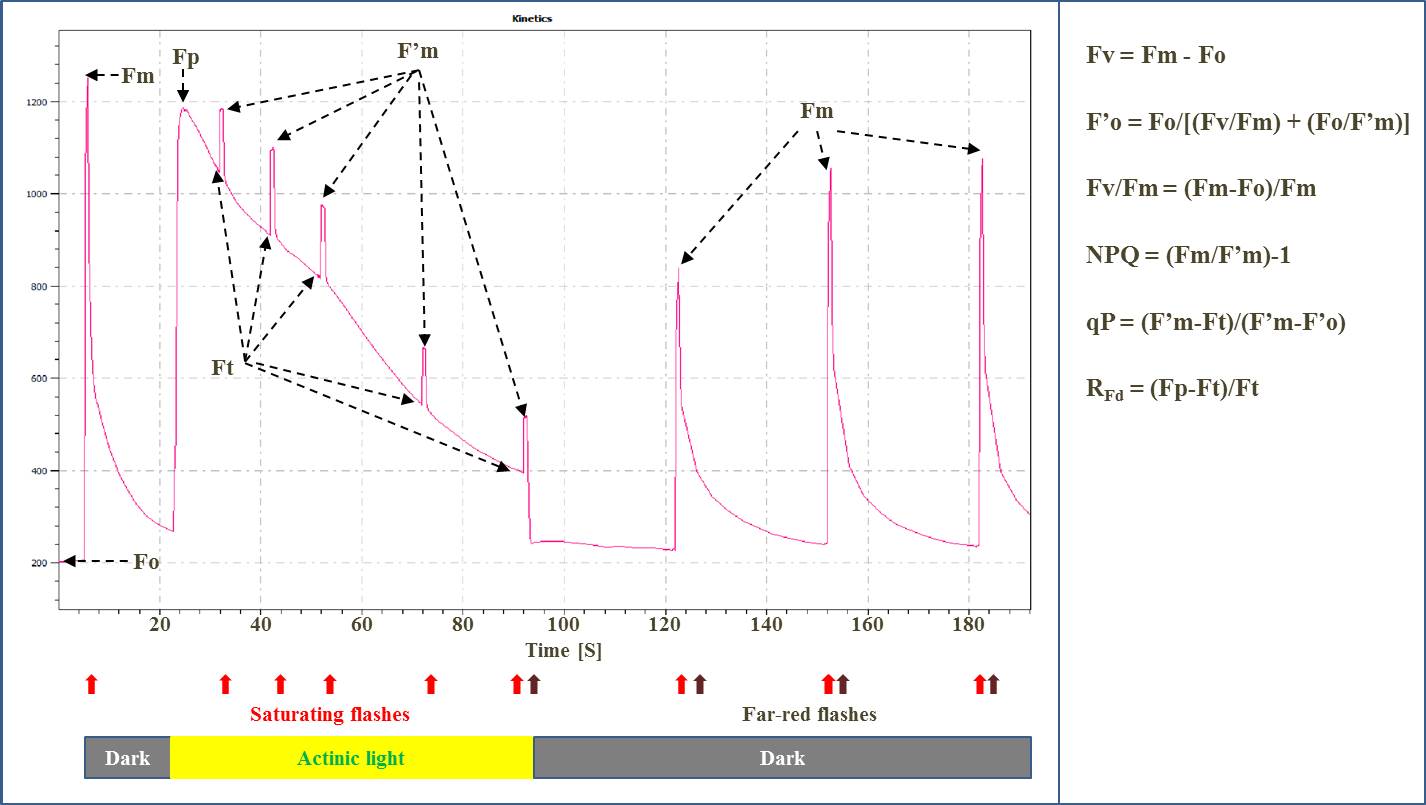
**

**SUPPLEMENTARY FIGURE S4.** Chlorophyll fluorescence induction kinetics measured in dark-adapted leaves with the PAM fluorometer. The minimum chlorophyll fluorescence F_o_ is induced by the weak measuring light, and the maximum chlorophyll fluorescence F_m_ is obtained by a saturating flash of white light. The chlorophyll fluorescence induction kinetics is induced by continuous actinic light, which is non-saturating, generating F_p_ that declines to the steady state level Ft. When actinic light is turned off, F_o_’ is obtained after an oxidation of the reduced PQ-pool using far-red flash. Saturating light flashes during the actinic light-induced kinetic show the maximum level F_m_’ in the light-adapted state of the leaf. From the kinetics chlorophyll fluorescence parameters are calculated and used to determine photochemical (qP) and non-photochemical quenching coefficients (NPQ) as well as maximum efficiency of PSII (Fv/Fm) and R_Fd_ that represent the quantum yield of PS II photochemistry and the variable chlorophyll fluorescence decrease ratio, respectively.
